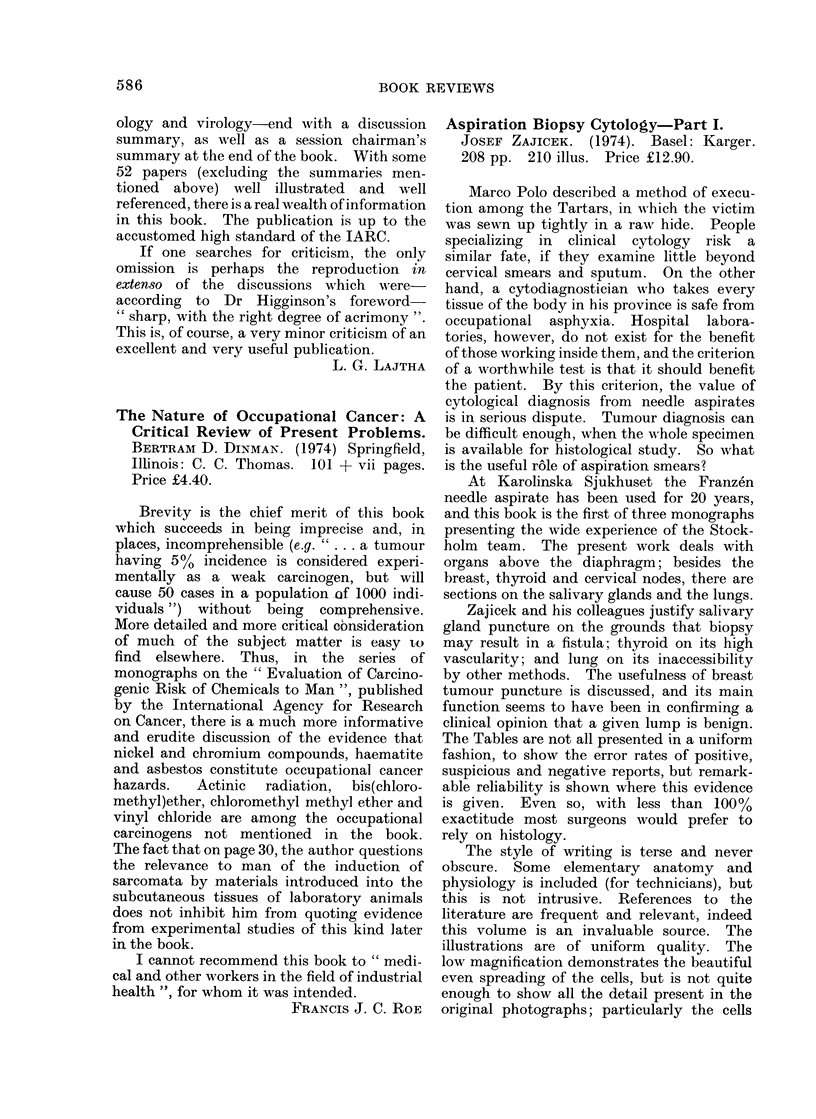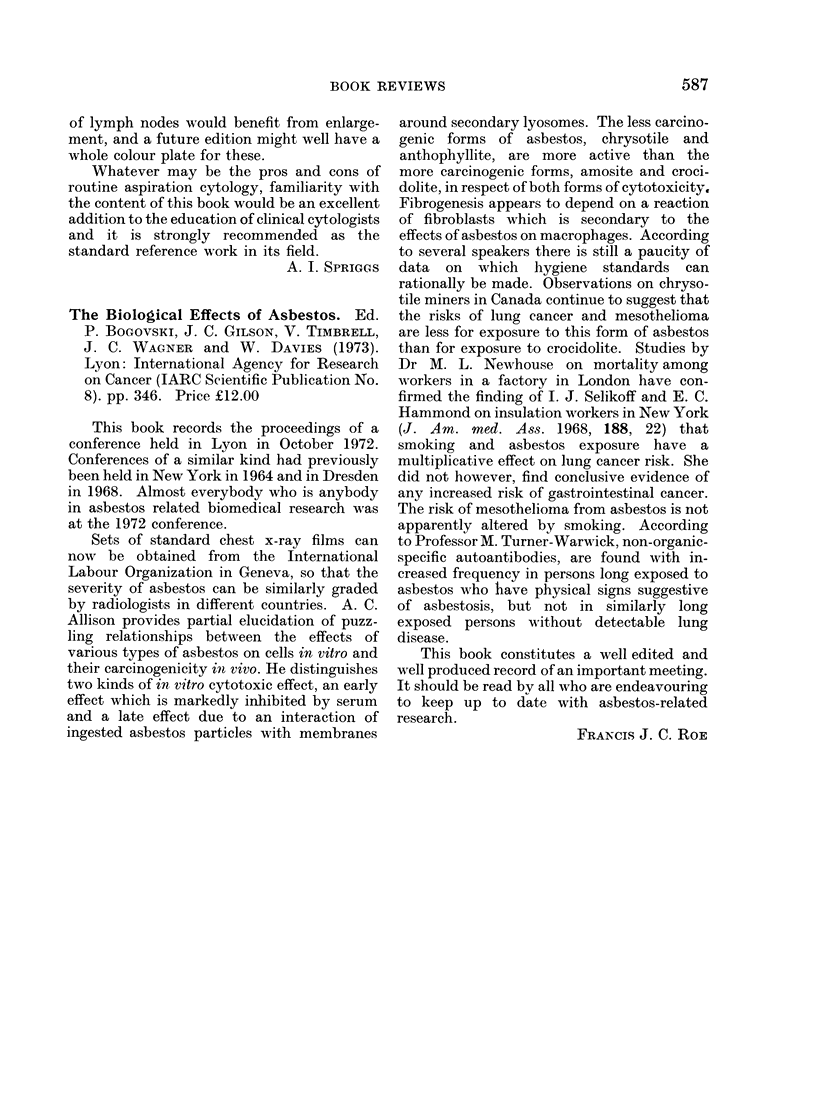# Aspiration Biopsy Cytology—Part I

**Published:** 1974-12

**Authors:** A. I. Spriggs


**Aspiration Biopsy Cytology—Part I.** JOSEF ZAJICEK. (1974). Basel: Karger. 208 pp. 210 illus. Price £12.90.

Marco Polo described a method of execution among the Tartars, in which the victim was sewn up tightly in a raw hide. People specializing in clinical cytology risk a similar fate, if they examine little beyond cervical smears and sputum. On the other hand, a cytodiagnostician who takes every tissue of the body in his province is safe from occupational asphyxia. Hospital laboratories, however, do not exist for the benefit of those working inside them, and the criterion of a worthwhile test is that it should benefit the patient. By this criterion, the value of cytological diagnosis from needle aspirates is in serious dispute. Tumour diagnosis can be difficult enough, when the whole specimen is available for histological study. So what is the useful rôle of aspiration smears?

At Karolinska Sjukhuset the Franzén needle aspirate has been used for 20 years, and this book is the first of three monographs presenting the wide experience of the Stockholm team. The present work deals with organs above the diaphragm; besides the breast, thyroid and cervical nodes, there are sections on the salivary glands and the lungs.

Zajicek and his colleagues justify salivary gland puncture on the grounds that biopsy may result in a fistula; thyroid on its high vascularity; and lung on its inaccessibility by other methods. The usefulness of breast tumour puncture is discussed, and its main function seems to have been in confirming a clinical opinion that a given lump is benign. The Tables are not all presented in a uniform fashion, to show the error rates of positive, suspicious and negative reports, but remarkable reliability is shown where this evidence is given. Even so, with less than 100% exactitude most surgeons would prefer to rely on histology.

The style of writing is terse and never obscure. Some elementary anatomy and physiology is included (for technicians), but this is not intrusive. References to the literature are frequent and relevant, indeed this volume is an invaluable source. The illustrations are of uniform quality. The low magnification demonstrates the beautiful even spreading of the cells, but is not quite enough to show all the detail present in the original photographs; particularly the cells of lymph nodes would benefit from enlargement, and a future edition might well have a whole colour plate for these.

Whatever may be the pros and cons of routine aspiration cytology, familiarity with the content of this book would be an excellent addition to the education of clinical cytologists and it is strongly recommended as the standard reference work in its field.

A. I. Spriggs